# Precautionary labelling of foods for allergen content: are we ready for a global framework?

**DOI:** 10.1186/1939-4551-7-10

**Published:** 2014-04-30

**Authors:** Katrina J Allen, Paul J Turner, Ruby Pawankar, Stephen Taylor, Scott Sicherer, Gideon Lack, Nelson Rosario, Motohiro Ebisawa, Gary Wong, E N Clare Mills, Kirsten Beyer, Alessandro Fiocchi, Hugh A Sampson

**Affiliations:** 1 Murdoch Childrens Research Institute, Department of Allergy and Immunology, The University of Melbourne, Melbourne, Australia; 2 Department of Paediatrics, Royal Children’s Hospital, Parkville, Australia; 3 Institute of Inflammation and Repair, Manchester Academic Health Sciences Centre, Manchester Institute of Biotechnology, The University of Manchester, Manchester, UK; 4 Section of Paediatrics, Allergy and Infectious Diseases, MRC and Asthma UK Centre in Allergic Mechanisms of Asthma, Imperial College London, London, UK; 5 Division of Paediatrics & Child Health, University of Sydney, Sydney, Australia; 6 Division of Allergy, Department of Pediatrics, Nippon Medical School, 1-1-5, Sendagi, Bunkyo-ku, Tokyo 113-8603, Japan; 7 Food Allergy Research & Resource Program, Department of Food Science & Technology, University of Nebraska, Lincoln, NE, USA; 8 Division of Allergy and Immunology, Department of Pediatrics, Jaffe Food Allergy Institute, Icahn School of Medicine at Mount Sinai, New York, NY, USA; 9 Division of Asthma, Allergy and Lung Biology, MRC and Asthma UK Centre in Allergic Mechanisms of Asthma, King’s College London, London, UK; 10 Children’s Allergy Unit, Guy’s and St. Thomas’ NHS Foundation Trust, London, UK; 11 University of Parana, Curitiba, Brazil; 12 Department of Allergy, Clinical Research Center for Allergy and Rheumatology, Sagamihara National Hospital, Tokyo, Japan; 13 Department of Paediatrics and School of Public Health, Chinese University of Hong Kong, Shatin, Hong Kong; 14 Department of Pediatric Pneumology and Immunulogy, Charité Universitätsmedizin Berlin, Berlin, Germany; 15 Hospital Bambino Gesù, Vatican City, Rome, Italy

**Keywords:** *Allergen labelling*, *Food allergy*, *Legislation*, *Precationary allergen labelling*, Anaphylaxis, Allergen avoidance, Mandatory labelling

## Abstract

Food allergy appears to be on the rise with the current mainstay of treatment centred on allergen avoidance. Mandatory allergen labelling has improved the safety of food for allergic consumers. However an additional form of voluntary labelling (termed precautionary allergen labelling) has evolved on a wide range of packaged goods, in a bid by manufacturers to minimise risk to customers, and the negative impact on business that might result from exposure to trace amounts of food allergen present during cross-contamination during production. This has resulted in near ubiquitous utilisation of a multitude of different precautionary allergen labels with subsequent confusion amongst many consumers as to their significance. The global nature of food production and manufacturing makes harmonisation of allergen labelling regulations across the world a matter of increasing importance. Addressing inconsistencies across countries with regards to labelling legislation, as well as improvement or even banning of precautionary allergy labelling are both likely to be significant steps forward in improved food safety for allergic families. This article outlines the current status of allergen labelling legislation around the world and reviews the value of current existing precautionary allergen labelling for the allergic consumer. We strongly urge for an international framework to be considered to help roadmap a solution to the weaknesses of the current systems, and discuss the role of legislation in facilitating this.

## Introduction

Avoiding specific foods and ingredients to which patients are allergic poses an important health challenge, especially in view of the increase in prevalence of food allergies in both developed and developing countries [1-8]. With the rising growth and development of worldwide manufacturing and more efficient ways to transport products at low cost around the world, what we now eat is increasingly provided by a food bowl that is global in scope (see Box 1). This has a significant impact on both food quality and safety, as different countries are governed by different manufacturing regulations and guidelines. There is a wide disparity between developed and developing countries with regards to the control and regulation of food labelling [9], and even among developed countries significant differences exist. This is of major importance to those with food allergies, who need to know with a high degree of certainty whether or not a food they consume contains an allergen, perhaps as a result of cross-contamination during production, and is therefore likely to trigger an adverse reaction. This paper aims to provide evidence regarding the variation in government guidelines and regulatory oversight of food labelling with particular focus on the vexed issue of precautionary allergen labelling. We discuss how a globally systematic and harmonized approach to food labelling will benefit consumers, regulators and manufacturers alike.

### Case Study

In December 2010/January 2011, at least 6 peanut-allergic individuals experienced significant allergic reactions after eating a variety of seafood products in geographically-distinct areas in Australia. The products all contained a crumb coating, supplied by a company in Beijing, China, which in turn contained soy flour supplied by a third party company. It is believed that the supply chain for the soy flour involved a number of companies, one of which had changed its production line resulting in contamination from peanut flour. This case demonstrates the difficulty in allergen-tracing along the supply chain and the increasing tendency to source raw ingredients from abroad. [http://allergenbureau.net/march-2011-news-round/].

### What are the various types of mandatory food labelling relating to food allergens?

In 1999, the World Health Organisation Codex Alimentarius Commission established guidelines for all countries that outlined the main 8 foods that should be considered for allergen labelling [10]. Subsequently, food labelling legislation has been introduced in many other countries, as outlined in Table 1. In general, allergen labelling can be divided into 2 categories:

1) **Labelling of allergens that are present in the ingredients and used in the production of the food.** Legislation mandating labelling of allergenic ingredients has now been introduced in a large number of countries (Table 1). Most developed countries mandate labelling of the most common allergenic foods such as peanuts, tree nuts, milk, eggs, fish, crustacea/shellfish, soy and wheat or cereals containing gluten, as well as ingredients derived from those foods in accordance with the 1999 Codex Alimentarius Commission guidelines. However, there are significant differences between countries as to what allergens are required to be disclosed, and the manner in which this is communicated to the consumer. Many countries recommend two methods, either highlighting the presence of an allergen in the ingredients list itself, or as a separate ‘contains X’ statement for allergenic ingredients. Within the European Union (EU) both methods are currently permitted but only the first is mandatory. From December 2014, the latter will be prohibited [26], presumably as a means to encourage consumers to study ingredients lists themselves rather than rely on optional non-mandatory statements which may be incomplete.

The list of specific allergens that require mandatory declaration often varies between countries. Most, but not all, include the 8 allergens proscribed by the Codex – Japan is a notable exception, where the pattern of food allergy is different from elsewhere and consequently, mandatory disclosure is limited to those allergens which are prevalent locally [18]. There are also differences in the definition of allergen categories, particularly tree nuts: within the EU, pine nuts are considered to be seeds, but are classified as a tree nut in USA and Canada. In the USA, several additional products are considered tree nuts, including coconut, shea nut and lychee. Some of these are not tree nuts botanically: coconut palms are not trees but ferns; lychee is a fruit and not a nut. The declaration of molluscs is also variable, with some countries classifying this as a ‘fish’ while in others, the inclusion is not clear (see Table 1).

The mandatory disclosure of food allergens is not exclusive to developed countries; a significant proportion of countries in Latin America now require mandatory labelling, including: Argentina, Bolivia, Brazil, Chile, Colombia, Costa Rica, Cuba, Mexico, Nicaragua and Venezuela. In the Far East and Asia, countries that have legislated allergen disclosure include Malaysia, South Korea, Japan, and Singapore. India is currently considering mandatory disclosure. China introduced legislation in 2012, perhaps in part due to the wide export of food products to countries where mandatory disclosure is required. The ‘policing’ and monitoring of allergen disclosure can vary from country to country (including ‘developed’ countries), and is poorly described in the literature.

In most countries with mandatory allergen labelling, disclosure is only required for pre-packed foods. From December 2014, legislation is being introduced throughout the European Union which will require the mandatory disclosure of allergens in non-pre-packed foods purchased for example from bakeries, butchers, and catering outlets, such as fast-food outlets and delicatessens [26]. Of note, the new legislation does not extend to potential contamination with allergens during production. However the requirement to manage potential contamination with a view to protecting allergic consumers is covered through European Commission Regulations 178/2002 and 852/2004. The legislation requires that manufacturers have to take account of the special needs of minority groups such as individuals with food allergies.

**Figure 1:**
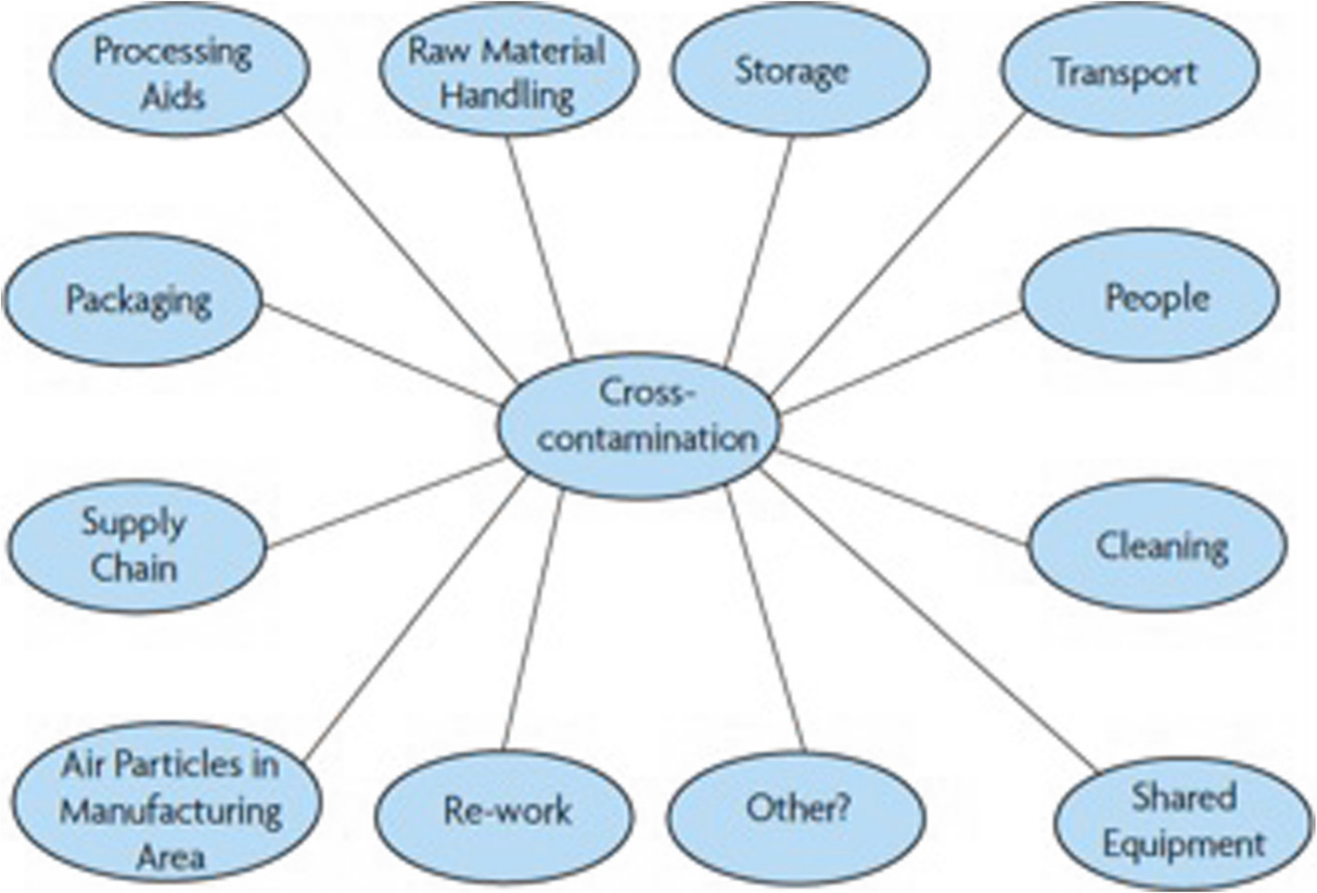
Potential sources of allergen contamination/cross contact during food production (Source: UK FSA).

2) **Precautionary statements relating to allergens which might be present due to cross-contamination during food production.** Foods can become contaminated with residues of allergenic foods at many points along the food chain (Figure 1), and different products may be produced on shared equipment, some containing allergenic ingredients and others not [27]. This may pose relevant health risks to those with food allergies. Uncertainty over the risk posed to allergic individuals by even very minute residual amounts/traces of allergen has prompted many food manufacturers to provide advice as to the potential for unintentional contamination with allergens during manufacture in the form of precautionary allergen labelling (PAL), also known as “may contain” statements. However, in the vast majority of countries, the use of PAL is not regulated by legislation (Table 2), and it is suspected that in many cases, a formal risk assessment is not performed to guide the use of PAL.

**Table 1:** Examples of countries with mandatory disclosure of allergens in pre-packed foods

	**Wheat**	**Other gluten-containing cereals**	**Egg**	**Milk**	**Peanut**	**Tree nuts**	**Soy**	**Fish**	**Crustacean**	**Mollusc**	**Celery**	**Mustard**	**Sesame**	**Lupin**	**Sulphur dioxide**	**Other**
Argentina [11]	✓	✓	✓	✓	✓	✓	✓	✓	✓						✓	✓^1^
Australia/ New Zealand [12]	✓	✓	✓	✓	✓	✓	✓	✓	✓	^2^			✓		✓	
Brazil [13]	✓	✓	✓	✓	✓	✓	✓	✓	✓						✓	✓^1^
Canada [14]	✓	✓	✓	✓	✓	✓	✓	✓	✓	✓		✓	✓		✓	
China [15]	✓	✓	✓	✓	✓	✓	✓	✓	✓							
European Union* [16]	✓	✓	✓	✓	✓	✓	✓	✓	✓	✓	✓	✓	✓	✓	✓	
Hong Kong [17]	✓	✓	✓	✓	✓	✓	✓	✓	✓						✓	
Japan [18]	✓	^3^	✓	✓	✓	^3^	^3^	^3^	✓^4^	^3^						✓^3^
Kuwait/Gulf [19]	✓	✓	✓	✓	✓	✓	✓	✓	✓				✓			
Malaysia [20]	✓	✓	✓	✓	✓	✓	✓	✓	✓							
Mexico [21]	✓	✓	✓	✓	✓	✓	✓	✓	✓						✓	
Singapore [22]	✓	✓	✓	✓	✓	✓	✓	✓	✓	✓					✓	
South Africa [23]	✓	✓	✓	✓	✓	✓	✓		✓	✓						
South Korea [24]	✓	^5^	✓	✓	✓		✓	^5^	✓^4^							✓^5^
USA [25]	✓		✓	✓	✓	✓^6^	✓	✓	✓						✓	
Codex [10]	✓	✓	✓	✓	✓	✓	✓	✓	✓							

Table adapted from http://farrp.unl.edu/IRChart with reference to national legislation.

*The 28 constituent member states of the European Union (EU) are: Austria, Belgium, Bulgaria, Croatia, Cyprus, Czech Republic, Denmark, Estonia, Finland, France, Germany, Greece, Hungary, Ireland, Italy, Latvia, Lithuania, Luxembourg, Malta, Netherlands, Poland, Portugal, Romania, Slovakia, Slovenia, Spain, Sweden, United Kingdom.

^1^ Local legislation also requires mandatory disclosure of tartrazine.

^2^ It is unclear whether disclosure of mollusc is required by local legislation.

^3^ Local legislation requires mandatory disclosure of eggs, milk, wheat, buckwheat, peanuts, shrimp and crab. In addition, disclosure is recommended (but not required) for the following 18 ingredients: abalone, squid, salmon roe, orange, kiwifruit, beef, walnut, salmon, mackerel, soybean, chicken, banana, pork, Matsutake mushroom, peach, yam, apple, and gelatin.

^4^ Legislation specifies prawn/shrimp and crab rather than ‘crustacea’.

^5^ Local legislation requires mandatory disclosure of egg, milk, buckwheat, peanuts, soybeans, wheat, mackerel (but not other finned fish), prawn/shrimp, crab, pork, peaches and tomatoes. There are no allergens for which labelling is optional.

^6^ Tree nuts in USA include a range of native nuts not included, for example, under EU legislation e.g. Beech, Butternut, Chestnut, Coconut, Ginko nut, Hickory nut, Lychee, Shea nut.

**Table 2:** Presence and regulation of additional/precautionary allergen labelling on prepacked foods

	**Precautionary allergen labelling**			**“Contains…” labelling permitted**	**Legislation on allergen disclosure implemented**
	**In use?**	**Is use regulated?**	**Risk-based approach, using thresholds?**		
Argentina [11]	NO	USE IS PROHIBITED	NO	YES and may be used as an alternative to precautionary labelling to indicate potential cross-contamination	2010
Australia/New Zealand[12]	✓	No	Voluntary. Thresholds vary with allergen	✓	2002
Canada [14]	✓ (specific phrasing recommended)	No	No	✓	1994
Chile [28]	✓	No	No	YES and can be used to indicate risk from cross-contamination. NB free-from labels prohibited	2010
China [15]	✓	No	No	✓	2012
European Union [16,26]	✓	No*	No	No longer permitted from Dec 2014	2003
Hong Kong [17]	✓	No	No	✓	2004
Japan [18]	NO	USE IS PROHIBITED	>10 ppm requires mandatory disclosure for all allergens	YES, only for allergen present in >10 ppm	2002
Kuwait/Gulf [19]	✓	No	No	✓	2008
Malaysia [20]	✓	No	No	✓	2009
Mexico [21]	✓	No	No	✓	2010
Singapore [22]	✓	No	No	✓	2011
South Africa [23]	✓	Yes**	No	✓	2012
South Korea [24]	✓	No	No		2004
Switzerland [29]	✓	Precautionary statements can only be use for non-ingredients above 1 g/kg	Any allergen (whether ‘ingredient’ or not) above 1000 ppm requires disclosure	✓	2002
USA [25]	✓	No	No	✓	2006

*Indiscriminate use of PAL might be construed as misleading and is therefore prohibited by EU legislation. However, no risk assessment is mandated prior to use of PAL therefore suspicion of any risk of contamination (however minimal) can be used to justify use of PAL.

**Legislation requires use of precautionary labelling to be substantiated by a documented risk assessment demonstrating adherence to GMP.

While PAL might be conceived as a useful strategy to convey risk of allergen cross-contamination, in practice their use has generated considerable uncertainty over their meaning [30]. A number of workshops have been held between different stakeholders, including industry, to drive good practice towards developing a standardised approach to allergen risk assessment and the use of PAL [31,32]. However, application of PAL remains inconsistent across industry and products, i.e. it does not represent a defined risk that can be communicated to consumers and other stakeholders. Furthermore, it is suspected that some manufacturers use PAL as an alternative to allergen risk management, rather than as a means to communicate the actual risk of cross-contamination following a risk assessment and intervention to minimise risk according to Good Manufacturing Practice (GMP).

### How common is the use of precautionary allergen labelling?

**Figure 2:**
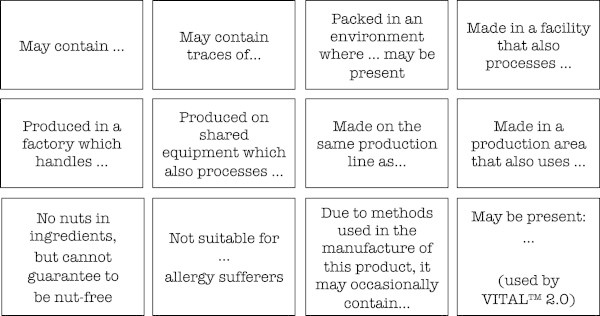
Examples of advisory warnings found on food labels.

Studies have shown a high prevalence of PAL. A survey commissioned by the UK Food Standards Agency (FSA) in 2001 assessed the prevalence of PAL in an ‘average’ shopping basket of 232 food items. The survey reported that 69% of cereals and 56% of confectionery items were labelled as containing ‘traces’ of nuts, despite none listing peanut or tree nuts as an ingredient [33]. A more extensive survey of over 20,000 unique products from 99 supermarkets in the United States found that 17% had PAL [34]. In the category of certain convenience foods, such as cookies and confectionery items, the rate exceeded 50%. The study also disclosed 25 different labelling terms used to indicate potential inclusion of the allergen, with PAL that could be classified into 3 broad categories: “may contain…”, “produced on shared equipment…” and “made in the same factory as…” (Figure 2). A similar observation has been made in a survey of 1355 supermarket products in Australia, where 65% of items included a precautionary statement of some sort [35].

### Do allergic consumers heed precautionary allergen labelling statements?

Avoidance of foods with PAL places an additional burden on the allergic consumer, with a survey reporting that shoppers avoiding products with PAL spending 39% more time identifying suitable foods, and paying on average 11% more than their non-allergic counterparts [33]. The widespread use of PAL causes considerable confusion and anxiety to people with allergies and their caregivers, and it is not uncommon for consumers to miss allergy warnings altogether [33,36,37]. The use of different wording on PAL statements is confusing and may contribute to the increasing trend for consumers to ignore them altogether [38,39].

A survey of young food-allergic adults in the USA in 2006 reported that over 40% ignored PAL [40]. Hefle et al. conducted a similar survey assessing consumer views prior to and after the introduction of new food labelling legislation in the USA, with over 600 individuals questioned during the 2003 and 2006 Food Allergy & Anaphylaxis Network conferences in the USA [39]. These participants might be expected to demonstrate increased concern over exposure to ‘trace’ amounts in foods with PAL, but surprisingly, 25% of participants in 2006 admitted to ignoring some PAL. The majority avoided foods labelled “may contain” but many assumed that statements such as “shared facility” implied a lower level of risk. A UK based survey found that 60% of parents of children with nut allergies avoided products labelled “may contain traces,” but only 40% did so when less direct statements were used – for example, “made in a factory that uses nuts” [41]. Similar findings have been reported in Japan [42], Canada [43] and Australia [44], suggesting that the more ambiguous the warning, the less likely consumers are to heed the content. Furthermore, there is evidence that consumers are ignoring PAL irrespective of whether they have a history of prior anaphylaxis – patients who might be expected to be more cautious with avoidance of foods with PAL [45].

Barnett et al. investigated the process through which 32 peanut- and nut-allergic adults interpreted PAL when purchasing food [46]. Only 3 (9%) were clear in their claim to judiciously avoid all foods with PAL. Many claimed such labelling was not credible or desirable, using 1 or more of 4 main strategies:

1) PAL is so prevalent that one cannot avoid eating food products with them

2) PAL was seen as being used to ‘protect’ the manufacturer from any claims arising from an allergic reaction due to cross-contamination

3) More ‘wordy’ PAL was interpreted as implying a lower risk, and could therefore be consumed by those who avoided products with more direct “may contain” labels.

4) Implausibility, when, for example, PAL to nuts appeared on a packet of peanuts, the implication being that PAL are not rationally or reliably applied by manufacturers.

Interpretation and consequent decisions were not only based on the detail of the labelling but also on external factors such as the nature of the product, any discounts on the product, the perceived trustworthiness of the producer and on the previous experience of the nut allergic individual (e.g. if they had already bought and consumed products with this type of labelling with no reaction). Some consumers would ignore the PAL if they ‘liked’ the food product and they felt that any consequential reaction would only be mild in severity.

Recent years have seen a growing awareness of these issues among the clinical community, although practice varies about the advice given to patients on whether foods with PAL are safe for them [47]. Clearly, health professionals charged with advising the allergic consumer are faced with the same issues as the consumer when interpreting the current situation. International efforts to define threshold levels of allergens along with validated allergen detection methods are essential to address these problems at a policy level.

### Does the use of precautionary allergen labelling correspond with risk of allergen exposure?

**Figure 3:**
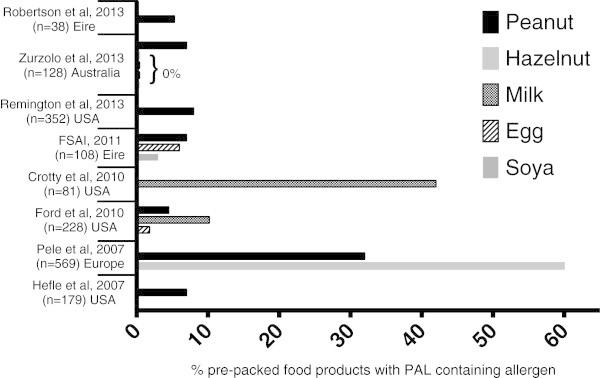
**Prevalence of allergen cross-contamination in prepacked foods with PAL.** (* Note: Zurzolo et al. also assessed products for milk, egg, hazelnut and soya; these allergens were not detected in any product [45]. The higher rates of allergen detection in surveys by Crotty et al. [48] and Pele et al. [49] are most likely due to the food products included: dark chocolate and cookie biscuits/chocolate respectively).

Relatively few studies have been conducted to evaluate the risks posed by allergen residues in pre-packed foods, and whether these correspond to the presence of PAL. The available data are summarised in Figure 3 from studies during the recent period 2006–2012 when improved allergen detection has become available [39,45,48-53]. These studies are subject to unavoidable methodological limitations, in that most did not assess batch-to-batch and within-batch variations in allergen content (with the exception of Zurzolo et al. [45]), and many only sampled a small number of the available products. Nonetheless, it is clear that the vast majority of foods with PAL did not contain evidence of allergen contamination.

Some studies also assessed the presence of allergen contamination in food products without PAL. Pele et al. analysed 544 food products (cookie biscuits and chocolates) for peanut content from 10 European countries in 2006 [49]. While 32% of products with PAL were found to have peanut contamination, 25% of items without PAL had evidence of peanut contamination. Ford et al. found allergen contamination (milk, egg, or peanut) in 5.3% of products with PAL compared to 1.9% of products without [53]. Notably, peanut was not detected in any of the 120 products tested that had no PAL. It is thus possible that the risk of peanut contamination is reducing over time as awareness within the food industry increases. In Ireland, 11% of 106 food products without PAL contained undeclared allergen, compared to 6.5% of 108 products with PAL [52]. Thus, the absence of PAL does not imply a food is safe for consumption by allergic individuals; this is poorly communicated to both allergic consumers and healthcare professionals alike.

Biochemical analysis of foods (mostly using ELISA based assays) have demonstrated that there is little correlation between the wording of the PAL and the risk of cross-contamination [39,48,49,51]; indeed, one USA study found that the rate of contamination was higher when the PAL stated “prepared in a shared facility” than when the label read “may contain” or “produced on shared equipment” [39]. However, differences in detection of allergens in such studies is confounded by variations in the quality of the analytical methods used which complicates interpretation of results [54]. Allergic consumers (and their families) may interpret ‘tolerance’ of food items with PAL as a sign of a more ‘mild’ food allergy, which can result in less stringent allergen avoidance. The widespread use of poorly defined labelling has resulted in a loss of credibility which might, paradoxically, lead to increased risk taking.

### What is the actual risk to the allergic consumer?

The risk to the allergic consumer in eating a product which may contain allergen due to cross-contamination is not solely related to the amount of allergen potentially present in that item. It also depends on the amount of the product consumed and the amount of allergen needed to trigger a reaction in that particular individual, known as an eliciting dose (ED). Data relating to the former can be estimated from national surveys of food consumption, while eliciting doses can be determined from published series of food challenges (known as population thresholds). However, many countries lack these data and the absence of thresholds relating to genetically diverse populations represents a clear deficit in the literature.

Studies involving double blind, placebo-controlled food challenges have demonstrated that allergic individuals react to a range of allergen amounts, with often a 4–5 log-fold difference between the ‘most’ and ‘least’ sensitive subjects [55], although the stopping criteria used to determine these reaction thresholds are not always consistent. This has improved of late with the use of internationally-agreed criteria based on objective symptoms [56,57]. The actual allergen dose to which individuals react varies with a whole host of poorly-understood factors, including the nature of the product in which the allergen is contained (known as the food matrix) and a range of factors specific to the individual (for example, intercurrent viral infections) [30]. Furthermore, the most sensitive individuals are often excluded from food challenges due to safety concerns. Nonetheless, using statistical modelling, it is possible to determine the ED01, ED05 and ED10 doses for specific allergens (these are the eliciting doses to trigger reactions in 1%, 5% and 10% of the allergic population, respectively), which can then be used in risk prediction.

This method was used in two reports published in 2013. The first, by the team at the Food Allergy Research & Resource Program in Nebraska, USA, used a probabilistic risk assessment which estimated the probability of a reaction in peanut-allergic consumers from nutrition bars with PAL to peanut in USA to be between 2 and 10 predicted reactions per 1000 eating occasions, although this was probably as over-estimate of the actual risk; this translates into 0.8–1.1 reactions per 100,000 peanut-allergic individuals per day [51]. A second report, by the same team, evaluated the prevalence of cross-contamination in Ireland, by assessing allergen content in 38 pre-packed foods with PAL, 92% of which were confectionery or snack items [50]. Two (5%) were found to have peanut contamination (a chocolate bar and a cereal bar); taking into consideration the level of contamination and serving size, this translated into an estimated risk of 2.6 predicted reactions per 1000 eating occasions. While this implies that the vast majority of food products with PAL to peanut will be tolerated by most peanut-allergic individuals, there is a small risk of allergic reactions to these foods.

Given that many allergic individuals do not heed PAL and consume foods with PAL [39-44], it is perhaps surprising how few reports there are in the literature of reactions attributed to an allergen present in foods due to cross-contamination. Very few studies have attempted to systematically investigate causes of accidental reactions in allergic individuals and there is a clear need for further data to be collected at the population level. Sheth et al. described 651 food-allergic patients on a Canadian registry who experienced an allergic reaction due to inadvertent allergen exposure, and reported on the patients’ opinions as to the cause of their reaction [37]. Almost half attributed the reaction due to a labelling-related issue: 8.7% of reactions were attributed to failure to heed a PAL, while 16.6% blamed the reaction on the absence of a PAL in the presence of presumed cross-contamination. However, there is little published data relating to allergic reactions due to cross-contamination that have been substantiated by biochemical analysis of the food in question [58,59], and it is therefore difficult to determine how common reactions due to unintentional cross-contamination are, in reality.

### Improving the utility of precautionary allergen labelling

Some countries have taken steps to reduce the variety of labelling that should be used as PAL. For example, in 2006, the UK Food Standards Agency produced a comprehensive guide to best practice that recommends a non-quantitative approach to determining risk of allergen cross-contamination [27]. Although the guideline recommends uniform wording of advisory warnings, the advice is voluntary and has done little to reduce the prevalence and variety of PAL currently used in the UK [57]. Under new EU legislation, to be introduced from December 2014, allergen disclosure will be clearer within ingredients lists and separate ‘contains’ statements will no longer be permitted [26], but PAL will continue to fall outside the remit of legislation. Canada modified food labelling legislation in 2012, and now requires allergens (as actual ingredients) to be declared using common names (e.g. milk, rather than “casein”) among other measures, which is consistent with legislation in Australia, EU and the USA. However, disclosure of allergens which may be present due to cross-contamination, (using PAL statements) remain voluntary and unregulated, although the regulatory authority now recommends that only a single phrase - “may contain:” - is used [14].

The use of PAL is currently regulated in 4 countries – Switzerland, Japan, Argentina and South Africa.

#### Switzerland

The first country to utilise a threshold to guide the use of PAL was Switzerland in 2000 [29]. No labelling is required at levels below 10 mg/100 g (100 ppm) gluten for cereals or 1 g/kg (1000 ppm) for other allergens. However, this equates to a protein level of 100 mg in a 100 g serving for non-gluten allergens, a level predicted to cause an allergic reaction in up to 50% of peanut-allergic individuals [55]. Unfortunately, there are no data assessing the impact of this threshold on the incidence of allergic reactions. The legislation in Switzerland (which does not belong to the EU) is otherwise similar to that of the EU, with the additional stipulation that while PAL are permitted, they can only be used with regards to allergen presence resulting from cross-contamination where that allergen has been shown to be present above these threshold levels.

#### Japan

In 2002, mandatory food allergy labelling became regulated under Japanese law [18]. At the same time the use of “may contain” statements was strictly prohibited; a threshold of 10 microgram protein/g food weight (10 ppm) was established, above which mandatory labelling for the above allergens is required, irrespective of whether that allergen was intentionally present as an ingredient or due to cross-contamination. Manufacturers are required to use specified methods (ELISA, PCR, Western blot) to determine the need for allergen disclosure although there is little data as to how this requirement is monitored. The presence of allergen in quantities below 10 ppm does not require disclosure. However, alternative declarations, such as “this product contains minute amounts of X” or “made in a factory that produces products that contain” are permitted, and may be used by manufacturers to alert the consumer as to potential allergen cross-contamination. It has been suggested that these labels may be used to reflect a higher quality of food to the consumer: seafood, for example, is widely perceived to have health benefits and so foods ‘produced in the same factory’ might indicate a product of superior quality to the Japanese public (R Crevel, personal communication).

The Ministry of Health, Labour and Welfare (MHLW) of Japan was the first government body to provide a definition of a ‘trace’ allergen amount requiring disclosure and to regulate this by law, through the use of allergen detection assays. In so doing the Japanese government has recognised that zero tolerance of allergenic foods may be unrealistic. Not only has the Japanese government set threshold limits for allergens in food, but they have limited the use of PAL with the aim of protecting the allergic consumer. The threshold of 10mcg/g was chosen on the basis that while the use of thresholds for the management of allergens could be of considerable value to all stakeholders, data were largely considered inadequate to derive them in the past [60]. As an alternative, it was felt that “if more than a few micrograms of protein weight per ml of food or a few micrograms of protein per gram of food are contained in a food, labelling of that allergen is necessary” on the basis that this might result in an allergic reaction [18]. This assumes, therefore, that amounts under these levels are unlikely to result in an allergic reaction. However, this may not be true, at least in theory. A threshold of 10mcg/g threshold means that in practice, an allergic consumer would need to eat 1 kg of a food product to be exposed to 10 mg of allergen, a serving size greater than that which would normally be expected. However, as can be seen in Table 3, the ED01 and even ED10 for some allergens are under 10 mg. For example, a hazelnut-allergic person could, in theory, expose themselves to a food containing less than 10mcg/g allergen (and thus have no allergen disclosure on the label); in this scenario, a serving of just 10 g could contain sufficient allergen to trigger an allergic reaction in 1 in 100 hazelnut-allergic individuals. Similarly, for cow’s milk, a threshold of 10mcg/g might not be protective for up to 1 in 10 milk-allergic children, a proportion which we consider to be significant. However, little is known of the rate of allergic reactions due to undisclosed allergens, industry compliance with the need for analysis or the impact the regulations has had on purchase habits and quality of life measures of the allergic consumer. This data would help to validate Japan’s decisions regarding mandatory and precautionary labelling.

**Table 3:** Ministry of Health, Labour and Welfare (MHLW) of Japan threshold limits for allergen disclosure, and how these relate to serving size and (European) population thresholds

	**Population threshold (mg protein)**[61-63]		**Minimum serving size not requiring allergen disclosure under Japanese legislation that could contain sufficient protein to cause a reaction in:**	
	**ED01**[61,62]**(mg protein)**	**ED10**[63]**(mg protein)**	**1% of allergic individual**	**10% of allergic individual**
Peanut	0.2mg	2.8mg	20g	280g
Cow’s milk	0.1mg	0.1mg (<3.5 yrs)	10g	10g (<3.5 yrs)
		5.3mg (>3.5 yrs)		530g (>3.5 yrs)
Egg	0.03mg	0.6mg (<3.5 yrs)	3g	60g (<3.5 yrs)
		20.4mg (>3.5 yrs)		2kg (>3.5 yrs)
Hazelnut	0.1mg	8.5mg	10g	850g
Soya	1.0mg	n/a	100g	n/a
Wheat	1.0mg	n/a	100g	n/a

#### Argentina

More recently, Argentina introduced legislation in 2010, which prohibited the use of all PAL [11]. Food labelling must be clear, thus an allergen is either ‘present’ or absent. Manufacturers appear to be permitted to use “Contains X” statements to list allergens which might be present due to cross-contamination, irrespective of whether the allergen is actually present or not.

#### South Africa

South Africa recently introduced legislation which permits the use of PAL, but manufacturers need to demonstrate the potential presence of allergen due to cross-contamination despite adherence to Good Manufacturing Practice (GMP) through a documented risk assessment; use of PAL is otherwise prohibited [23].

### Is there an alternative to legislation and regulation?

The Voluntary Incidental Trace Allergen Labelling (VITAL) initiative developed by the Australian food industry’s Allergen Bureau represented a first attempt to introduce a formal and transparent basis for of risk assessment by manufacturers in the application of PAL [64]. The VITAL process was developed with the intent of replacing all other forms of precautionary labelling, using a validated risk assessment tool to determine the need for precautionary labelling.

Under VITAL, the manufacturer performs a risk assessment using the VITAL calculator which has been designed to alert the manufacturer of the possible presence of sufficient levels of allergen residues arising from cross contact and based upon analysis to provoke allergic reactions in consumers ingesting specified quantities of these products [65]. Once the manufacturer receives the raw material, the product information form (PIF) which provides specification of other information from the supplier for each ingredient is reviewed and a decision is made for each cross contact allergen. The manufacturing line and environment are then reviewed to determine if there are any cross contact allergens which may become incorporated in the product in the manufacturing process. The VITAL calculator then determines the final allergen content and compares this to Reference Doses which were defined for major allergenic foods. The initial VITAL action levels were based on minimum eliciting doses for regulated allergenic foods (expressed as doses of protein) collated by the 2006 U.S Food & Drug Administration (FDA) Threshold Working Group [66]. Initially, due to limited data on minimum provoking doses existing at that time, a 10-fold uncertainty factor was applied by VITAL to assure that sufficiently conservative action levels were promulgated. VITAL was recently revised (VITAL 2.0) following an expert panel review of threshold data [62], and no longer incorporates an uncertainty factor as the thresholds selected are deemed to be tolerated by 95 - 99% of the allergic population (see Table 3). There are now two actionable levels: if an item falls within Action Level One it requires no precautionary statement and the food is regarded as safe to eat by the allergic consumer. If the product falls within Action Level Two, VITAL “may be present” statement is used. VITAL Action Level Two is used for allergens in particulate form (a separate and distinct particle of material e.g. sesame seed). If present in the final product as a readily dispersible allergen (a powder or liquid in a homogenous form) the total protein concentration from the allergen source is determined and labelled according to mandated regulations. Importantly, under VITAL, only a single distinct precautionary statement – “may be present” – is to be used, to avoid the confusion which has resulted from multiple phrases used in PAL. The aim was that the appearance of this statement would imply that a VITAL assessment had been performed.

While interest from industry has been enthusiastic, actual implementation of the process has been more limited to date [35], which might be seen as a consequence of the voluntary nature of the scheme. Another shortcoming is that not all of the foods which have been subject to the VITAL assessment are identifiable to the consumer. Products that have been through the VITAL process and are found to be Action Level one do not carry a label and therefore an allergic consumer cannot distinguish that this food product is likely to be safer than one that has no label but not subjected to VITAL. This is currently the greatest limitation of VITAL 2.0 with respect to usefulness to the consumer as in essence it is an educational tool for manufacturers to ensure appropriate allergen risk management. Furthermore the measurement of actual allergen levels are not required as part of VITAL, although when contamination is suspected over and above that of Action Level two manufacturers usually send samples for analytical assessment and to reassess the manufacturing process and risk of contamination. While validation of the assessment can be performed through analysis of the food products for allergen cross-contamination, this is not mandatory due to the inherent limitations of allergen detection [54]. Importantly, VITAL and indeed all PAL does not cover the occurrence of an accidental contamination with large pieces of particulate matter such as a whole nut.

### Limitations of allergen detection methods for use by manufacturers

Controversies remain regarding the most valid and precise form of allergen detection [54]. Unfortunately the more reliable and precise the methodology the more likely it is to be cumbersome and cost-prohibitive and as such manufacturers often rely on less validated kit methodologies. Similarly, public health agencies in various countries have not provided guidance to industry on preferred approaches and as yet there is no universally-agreed method for allergen detection.

Sampling strategies remain another area of concern and uncertainty. The allergen residues in homogeneous foods, such as ice cream, are more likely to be uniformly distributed and sampling is a lesser issue. In other cases such as with particulate contamination especially, the number and selection of samples becomes critical to improve reliability for detecting allergen contamination [67]. Due to the uneven distribution of the allergen the ability to detect allergen becomes extremely variable. As yet there are no currently agreed industry standards on sampling for particulate contamination.

### Is there a role for legislation in establishing a global system for precautionary allergen labelling?

Establishing a system for PAL which is globally accepted and used widely means not only that industry itself can improve its practices and therefore food safety, but also provides the opportunity for better communication and management of allergen risks by clinical practitioners and their patients and thereby improves allergy management and food choices. However, should this be done through a legislative approach (as in Japan) or through voluntary measures? The 2006 guidance released by UK Food Standards Agency on best practice with regards to PAL had minimal impact on the utility of such labelling in the UK [57]. Indeed, in a subsequent evaluation of the 2006 guidance, almost two thirds of enforcement officers and large food producers surveyed felt the guidance should be compulsory [68]. While regulation may be tempting, there are a number of drawbacks to this. Legislation requires certainty – for example, legislation based on allergen analysis must consider the inherent problems in allergen detection (discussed above), and the need to use an appropriate detection method for different forms of the allergen (and where no appropriate allergen detection method, nor reference material exists) [54]. Significant research gaps remain in both the robustness of allergen detection techniques and clinical reactivity thresholds, as reviewed elsewhere [69]. Regulation may also result in a lack of flexibility to adapt to new methods of allergen detection and even new information relating to allergen thresholds as more data become available. These drawbacks need to be balanced against a possible need to use regulation to drive improvements in food allergen labelling.

**Figure 4:**
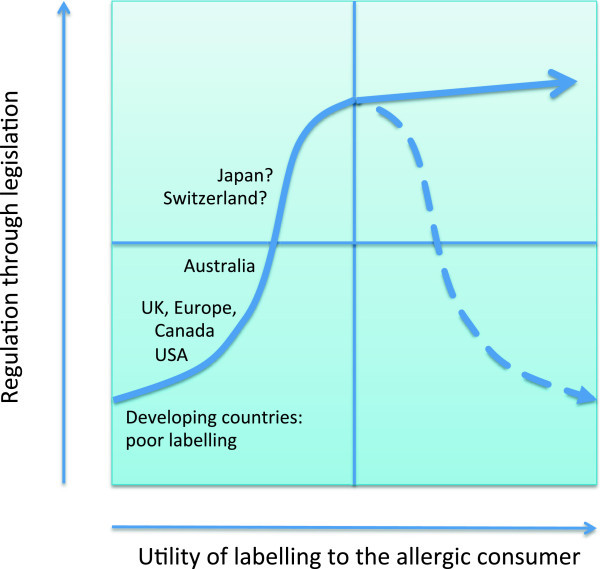
**Can regulation improve precautionary advisory labelling?** Legislation may be required to introduce uniformity amongst manufacturers in conducting a risk assessment for allergen content and then communicating that information to the allergic consumer. However, regulation has its weaknesses and needs to be balanced against the ability of a system to adapt to new technologies (e.g. in allergen analysis) and clinical data (e.g. updated allergen thresholds). Once established, ongoing improvements to PAL may be best achieved through a less rigid legislative process (depicted by the dotted line in the figure).

In this article, we have reviewed the current situation in many countries, which does not benefit either the allergic consumer or food manufacturers who are potentially liable for an allergic reaction resulting from cross-contamination. Those countries which have introduced legislation based on allergen thresholds (i.e. Japan and Switzerland) with regards to PAL may have improved the usefulness of such labelling to the allergic consumer, but further data are needed to substantiate this. We suggest that legislation will be required to introduce uniformity amongst manufacturers in conducting a risk assessment for allergen content and then communicating that risk in an easily understandable way to the allergic consumer. However, regulation through the legal system also has its weaknesses and therefore needs to be balanced against the ability of any system to communicate risk of allergen cross-contamination to adapt to new technologies and clinical data. This may best be achieved in the long run through a balance between self-regulation and manufacturing legislation (Figure 4). With the growing burden of food allergies it is clear that food labelling should become a priority amongst policy makers and health authorities.

Importantly, improved determination of reliable thresholds would better inform calculation of risk and therefore remain priorities for further research. Until now allergen threshold predictions have relied on data from oral food challenges that have employed protocols where up-dosing at regular intervals occurs until a single serve equivalent is tolerated or an allergic reaction intervenes. Although food challenges are the gold standard for the diagnosis of IgE-mediated food allergy, the precision around which dose the patient has reacted to is diminished because the intervals between up-dosing are usually 15–30 minutes for practical reasons – i.e. to minimise the impact on time spent doing the challenge when up to 7 doses need to be administered. As such it is not always clear whether the patient is reacting to the most recent dose, a preceding dose or to an overall cumulative dose. To counteract these limitations there are several single dose studies in preparation or underway to better delineate the lower values of the threshold curves. Zurzolo et al. [70] have initiated a multicentre trial in peanut allergic children to confirm or refute the currently determined ED05 (eliciting dose for 5% of the peanut allergic population). Studies such as these will help inform more reliable threshold predictions which will enable manufacturers to better predict the safety of their food.

### Are we ready for a global framework with regards to precautionary allergen labelling?

Precautionary allergen labelling remains a vexed issue for allergy consumers and clinicians alike. There is clearly an unmet need with regards the value of current PAL standards and utility and a clinical imperative to remove the burden of risk from the allergic consumer. A globally agreed framework would enable progress to be made to ensure that foods produced around the world can be safely ingested by consumers irrespective of allergy status. A globally relevant framework would enable careful consideration of issues such as should manufacturers universally implement risk assessment tools (e.g. VITAL 2.0 as in Australia) or whether governments should consider setting mandated thresholds and ban precautionary allergen labelling (as in Japan). Could such a framework embrace the differential complexities of manufacturing in developed versus developing countries and the potential difference in threshold levels for different populations? Could a global framework assist in engendering consensus on the most reliable and cost-effective allergen detection assays for international application? At the very least, international consistency of the types of statements employed for PAL would be a significant early step forward for improved utility and safety for the allergic consumer. Harmonisation of allergen regulations across the world will be important to both protect allergic consumers and help support effective trade in the global market place.

## Competing interests

KJA is a Viertel Senior Medical Research Fellow and received funding from the NHMRC and Australian Egg Corporation and has received speaker’s fees from Abbott, Danone, Wyeth and Pfizer, Nutricia, and Alphafarm. PJT is in receipt of a UK Government Medical Research Council Clinician Scientist award. RP declares she has no competing interests. ST receives royalties from a licensing agreement with Neogen Corp., Lansing Michigan USA for the sales of immunoassay kits for the detection of allergen residues in foods based upon antisera that were created in his laboratory. SS declares he has no competing interests. GL declares funding to his institution from the Immune Tolerance Network supported by the National Institute of Allergy and Infectious Diseases, the Food Allergy Research & Education (FARE), National Peanut Board, Food Standards Agency, MRC Athma UK Centre, Department of Health via the National Institute for Health Research comprehensive Biomedical Research Centre aware to Guy’s & St. Thomas’ NHS Foundation Trust in partnership with King’s College London and King’s College Hospital NHS Foundation Trust. GL has received monies from DBV Technologies. NR declares he has no competing interests. ME declares he has no competing interests. GW declares he has no competing interests. ENCM has funding, via the University of Manchester, from the UK Technology Strategy Board relating to development of allergen analysis methods with industrial partners including Waters Corporation LGC Ltd and Romer Diagnostics, UK Biological and Biotechnological Sciences Research Council funded studentships for allergen analysis in collaboration with industry, specifically Campden BRI, Waters Corporation and Genon. KB has received consulting or speaker’s fees from Danone, MedaPharma, ALK, Novartis, Unilever, Allergopharma, MedUpdate, HAL, Novartis, and funding from the European Union, German Research Foundation, ThermoFisher, Danone, DST and the Foundation for the Treatment of peanut allergy. AF declares he has no completing interests. HAS has received consulting fees and is a member of the Danone Scientific Advisory Board, has received speaker’s fees from ThermoFisher Scientific, UCB and Pfizer, and funding from the NIH and FARE. The authors declare that they have no competing interests.

## Authors’ contributions

KA and PJT contributed equally to the writing of the manuscript. The initial draft was then revised by KA, PJT, RP and HS, prior to subsequent review by the other authors. All authors have reviewed and approved the final version of manuscript.
